# Learning to experience side effects after antidepressant intake – Results from a randomized, controlled, double-blind study

**DOI:** 10.1007/s00213-016-4466-8

**Published:** 2016-11-02

**Authors:** Julia Rheker, Alexander Winkler, Bettina K. Doering, Winfried Rief

**Affiliations:** Division of Clinical Psychology and Psychotherapy, Department of Psychology, Philipps-University Marburg, Gutenbergstraße 18, 35032 Marburg, Germany

**Keywords:** Side effects, Nocebo, Antidepressants, Learning

## Abstract

**Background:**

Side effects play a key role in patients’ failure to take antidepressants. There is evidence that verbal suggestions and informed consent elicit expectations that can in turn trigger the occurrence of side effects. Prior experience or learning mechanisms are also assumed to contribute to the development of side effects, although their role has not been thoroughly investigated. In this study, we examined whether an antidepressant’s side effects can be learned via Pavlovian conditioning.

**Methods:**

Participants (*n* = 39) were randomly allocated to one of two groups and were exposed to a classical conditioning procedure. During acquisition, 19 participants received amitriptyline and 20 participants received a placebo pill. Pills were taken for four nights together with a novel-tasting drink. After a washout phase, both groups received a placebo pill together with the novel-tasting drink (evocation). Side effects were assessed via the Generic Assessment of Side Effects Scale prior to acquisition (baseline), after acquisition, and after evocation. A score of antidepressant-specific side effects was calculated.

**Results:**

Participants taking amitriptyline reported significantly more antidepressant-specific side effects after acquisition compared to both baseline and the placebo group. After evocation, participants who underwent the conditioning procedure with amitriptyline reported significantly more antidepressant-specific side effects than those who never received amitriptyline, even though both groups received a placebo.

**Conclusions:**

Our results indicate that antidepressant side effects can be learned using a conditioning paradigm and evoked via a placebo pill when applied with the same contextual factors as the verum.

## Introduction

The prescribing of antidepressants has risen over recent years, with up to 13.4 % of individuals in Western countries having been prescribed antidepressant medication at least once per year (Sihvo et al. 2010; Lockhart and Guthrie 2011; Mojtabai and Olfson 2014; Abbing-Karahagopian et al. 2014). Although antidepressants are effective in treating major depression (Cleare et al. 2015), patients often discontinue drug intake (Sawada et al. 2009; Bocquier et al. 2014). Rates of reported non-adherence vary, but some studies report rates of discontinuing antidepressant medication of over 50 % within the first 2 to 4 months (Serna et al. 2010), while others report even higher discontinuation rates (Bocquier et al. 2014). These rates are alarming, considering that guidelines suggest taking antidepressant medication for at least 6 to 9 months to prevent relapse after the remission of a depressive episode (Cleare et al. 2015). Several factors contributing to patients’ non-adherence have been identified (Serna et al. 2010; De las Cuevas et al. 2014; Bocquier et al. 2014), but one particular factor emerges consistently as a reason for discontinuing antidepressants, namely, side effects (e.g., Serna et al. 2010; Hung et al. 2011; Murata and Kanbayashi 2012; De las Cuevas et al. 2014). Common side effects of antidepressants (i.e., pharmacological reactions due to drug intake that differ from those intended) are, for instance, daytime sleepiness, dry mouth, loss of interest in sexual activity, and weight gain (Ashton et al. 2005).

Some of the adverse events occurring after medication intake can be attributed to the drug’s specific pharmacological action, and many such events are considered to be dose dependent, whereas others, not attributable to the drug’s pharmacological action, often appear to be dosage independent (Shedden Mora et al. 2011). The latter events can be studied in placebo groups in drug trials. All side effects occurring after the intake of an inert substance are not specific or attributable to the drugs’ pharmacokinetics (Schedlowski et al. 2015). The occurrence of side effects after placebo intake is called the nocebo effect. Originally, it was assumed that some nocebo side effects occur due to the misattribution of pre-existing symptoms (Barsky et al. 2002). More recent studies have additionally shown that the adverse effects occurring in placebo groups in drug trials match the side effects reported in the active drug arms of these trials (e.g., Rief et al. 2009; Amanzio et al. 2009; Mitsikostas 2012).

One explanation for the nocebo phenomenon is patients’ expectations about possible side effects in general (Nestoriuc et al. 2010), which might be triggered by the information provided in the informed consent or by verbal suggestion (Mondaini et al. 2007; Cohen 2014). Another factor potentially contributing to the occurrence of side effects is prior experience or learning (Amanzio 2015). One such example is cancer patients experiencing nausea as a side effect after undergoing chemotherapy. It is assumed that initially, neutral stimuli such as the room in which the therapy is administered are associated with the occurrence of nausea; therefore, just entering the room can cause anticipatory nausea after a while (Matteson et al. 2002). Such conditioning effects can be generated if an originally neutral stimulus (NS) (e.g., the room) is combined with an active stimulus (unconditioned stimulus (UCS)) (e.g., chemotherapy) that leads to certain reactions (e.g., nausea). After several pairings of NS and UCS (acquisition phase), the NS becomes a conditioned stimulus (CS). This means that the CS alone can evoke the reaction that was originally generated by the UCS (evocation; Pavlov 2010). Although some authors differentiate between expectations and conditioning as different mechanisms involved in placebo and nocebo responses (Enck et al. 2013), it is not always possible to clearly distinguish them since learning also leads to certain expectations (Stewart-Williams and Podd 2004). Therefore, in this article, we do not differentiate between expectation and conditioning per se but rather between “expectation through verbal suggestion” and “learning/conditioning.”

Learning effects have been experimentally investigated, showing, for example, that with motion sickness, a nocebo response can be learned (Klosterhalfen et al. 2009). Colloca et al. (2008) found in a conditioning paradigm that a light paired with a noxious stimulus can induce a hyperalgesic nocebo effect in the evocation trial. In a subsequent study using a similar paradigm, they showed that even one acquisition trial suffices to induce nocebo effects, although effects are more stable after additional trials (Colloca et al. 2010). Conditioned nocebo effects can also be evoked by non-conscious stimuli (Jensen et al. 2012).

When it comes to pharmacological responses, the role of conditioning has been demonstrated in conjunction with immune reactions (Albring et al. 2012). However, to the best of our knowledge, there has been no evidence forthcoming that reveals whether Pavlovian conditioning contributes to the development and maintenance of antidepressant side effects.

We hypothesized that participants taking amitriptyline would report more antidepressant-specific side effects in all after four nights of medication intake (acquisition phase, i.e., pill intake combined with a novel-tasting drink as NS) and attribute more of these side effects to the medication intake than would participants taking a placebo. Furthermore, we hypothesized that after having undergone the aforementioned acquisition and a subsequent washout phase, receiving a placebo pill together with the novel-tasting drink (evocation) would lead to more reported antidepressant-specific side effects in total and more medication-attributed antidepressant-specific side effects in the group that had previously taken amitriptyline than in the placebo group.

## Methods

### Participants and ethics

This study was conducted in the Division of Clinical Psychology at the Philipp University of Marburg in 2014. Participants aged between 18 and 69 years who were willing to refrain from alcohol consumption and driving during the study period were recruited via an advertisement at the university. To ascertain that only physically and mentally healthy participants were included, all subjects underwent a medical and psychological examination (by a study physician and a psychologist, both trained in Good Clinical Practice). These included interviews about medical history and mental health (according to the International Diagnosis Checklists; Hiller et al. 2004), an electrocardiogram, blood tests, and a urine pregnancy test (only in females). If the examinations yielded evidence of contraindications to the study medication as mentioned in the information sheet for health professionals, those participants were excluded.

Prior to the beginning of the study, participants were informed about the study design and treatment by the study physician. Written informed consent was obtained from all individual participants included in the study. The experiment was conducted according to the Declaration of Helsinki. Since the current study was only an exploratory subinvestigation in addition to the main study (for detailed results, see Winkler et al. 2016), only the main study was registered at http://www.clinicaltrials.gov (NCT02127736). Nevertheless, the outcomes used in this study were determined as secondary outcome measures in the study protocol, which was approved by the ethics committee of the medical chamber of Hessen (Landesärztekammer Hessen; FF51/2013). Participants were paid for study participation.

### Experimental design

**Fig. 1:**
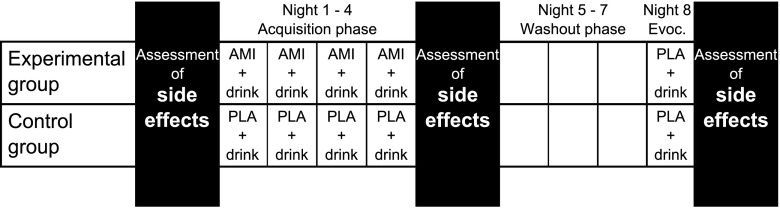
Experimental design

After the medical and psychological examination, equal numbers of participants were randomized into the placebo and antidepressant groups; no stratification was conducted. Randomization was done by an independent researcher. Through randomization, each individual got a number, which was assigned to a medication container which held either placebo or antidepressant pills. Both experimenters and participants were blinded to group allocation. The experimental group received amitriptyline; the control group received identical-looking placebo pills. At the baseline assessment, all subjects filled in the Generic Assessment of Side Effects Scale (GASE; Rief et al. 2010). Afterwards, participants in the experimental group underwent a classical conditioning paradigm (see Fig. 1). During the acquisition phase (nights 1 to 4), participants received 50 mg of amitriptyline (US) together with 100 ml of a novel-tasting drink that consisted of lychee juice with woodruff syrup and blue food coloring. The drink was the neutral stimulus (NS), which was supposed to become the CS. The drinks’ ingredients were chosen in order to increase the novelty, saliency, and distinctiveness of the CS, since it has been argued that this might increase the conditioned response (Doering and Rief 2012). Amitriptyline-neuraxpharm 50 mg was used and encapsulated for study purposes by licensed pharmacologists. To make pill intake more salient, the novel-tasting drink was used. Participants were instructed to take the medication and the novel-tasting drink immediately before going to bed on four subsequent nights. Once the acquisition phase was over, side effects during acquisition were assessed. The acquisition was followed by a 3-day washout phase (nights 5 to 7). On night 8, the evocation night, all participants received a placebo pill together with the novel-tasting drink (CS). The next day, side effects after evocation were assessed.

The placebo control group underwent the same procedure as the experimental group but received placebo pills instead of amitriptyline during the acquisition phase.

### Measures

Side effects were assessed with the GASE (Rief et al. 2010). The GASE contains a list of 36 symptoms and covers the most frequently reported side effects in clinical trials using different drugs according to FDA. The patient gives a rating for the presence and severity of each of these symptoms on a 4-point Likert scale ranging from “not present” (0) to “severe” (3). In addition, the patient indicates for each symptom whether he or she thinks it is caused by the intake of the drug (yes/no). A total score can be calculated as a sum of all item answers (general symptom load) as well as a total score of only medication-attributed symptoms. The GASE reveals good internal consistency with Cronbach’s *α* = 0.89 and has been validated in a large sample with more than 2500 participants (Rief et al. 2010).

#### Primary outcome measure

For the purpose of our study, an Antidepressant Composite Score (GASE-AD) was calculated to assess side effects specific for the study’s antidepressant. To assess the most frequently reported side effects, we chose items that at least 50 % of the experimental group had experienced after the acquisition phase. This criterion left us with four items: (1) dry mouth, (2) dizziness, (3) cardiovascular problems, and (4) fatigue or loss of energy. These symptoms are also listed in the Physician’s Desk Reference (Barnhart 1988) and in the Compendium of Psychiatric Pharmacotherapy (Benkert and Hippius 2014) as common symptoms of amitriptyline. In addition, these 4 symptoms are listed among 12 very common symptoms of amitriptyline on http://www.pharmawiki.ch (2015). We then calculated the score of all reported antidepressant specific side effects (GASE-AD) and that of all medication-attributed antidepressant specific side effects (GASE-AD-MA). Detailed analyses of potentially positive placebo effects are reported elsewhere (Winkler et al. 2016).

#### Further analyses

In addition to studying antidepressant-specific side effects, we analyzed more generic side effects or symptoms also, since symptoms not specific to the drug under investigation can also occur after taking a placebo pill (Barsky et al. 2002). For this purpose, the four antidepressant-specific items were excluded from calculating the scales for all reported generic, i.e., not antidepressant-specific side effects (GASE-generic) and for all medication-attributed generic side effects (GASE-generic-MA). To give a complete overview of reported side effects, we also analyzed the complete GASE scale (GASE-total) and calculated a score for all common side effects of amitriptyline (GASE-AMI) independent of how often they were named in the experimental group after acquisition. This score contains the items that are mentioned as the most frequently reported side effects both in the Compendium of Psychiatric Pharmacotherapy (Benkert and Hippius 2014) and on http://www.pharmawiki.ch. The items in the score are (1) dry mouth, (2) dizziness, (3) cardiovascular problems, (4) fatigue or loss of energy, (5) palpitations or irregular heartbeat, (6) constipation, (7) abnormal sweating, and (8) tremor. Weight gain was not included in the score since participants only took amitriptyline for 4 days. In addition, accommodation problems were also not included in the score because it was not assessed in the GASE. For the GASE-total and the GASE-AMI, medication-attributed scores were calculated as well (GASE-total-MA and GASE-AMI-MA).

To assess whether participants were unblinded by amitriptyline’s experienced side effect profile, we asked the participants after the acquisition phase to guess which experimental group (amitriptyline vs. placebo) they belonged to. After study completion and unblinding, this rating (perceived group allocation) was correlated with current group allocation.

To analyze any clinical correlates with the nocebo response, we applied the subscales of the Symptom Checklist-90-Revised (SCL-90-R; Derogatis 1994) and the Beck Depression Inventory (BDI; Beck et al. 1961), which were assessed at baseline and correlated those with the GASE-AD and GASE-AD-MA.

### Statistical analyses

We calculated the sample size with G*power (Faul et al. 2007). The initial sample size for the study was calculated for the primary outcome in the main study (Winkler et al. 2016); hence, only 40 participants were recruited. However, for the current investigation, the sample size was calculated post hoc and revealed that in order to detect a large time × group interaction effect with a power of 80 % and an *α* level of 0.05, the estimated total sample size was *n* = 42.

Statistical analyses were performed with IBM SPSS Statistics 21.0. Baseline characteristics were analyzed using *t* tests and *χ* ^2^ tests. Missing values in the GASE were replaced by multiple imputation.

To test for differences in total side effect reporting and medication-attributed side effect reporting between and within groups, multivariate analyses of variance (MANOVA) for repeated measures with the factors time (baseline, acquisition, evocation) and group (amitriptyline or placebo) were applied. Significant effects in the MANOVA were followed up by pairwise comparisons. The pairwise comparisons were adjusted according to the Bonferroni’s procedure; i.e., the within-group tests were adjusted for three comparisons each. The correlation between current group allocation and perceived group allocation was calculated via the phi coefficient. Correlations between the SCL-90-R subscales and the BDI and the GASE scales were calculated using the Pearson’s correlation coefficient.

## Results

**Fig. 2:**
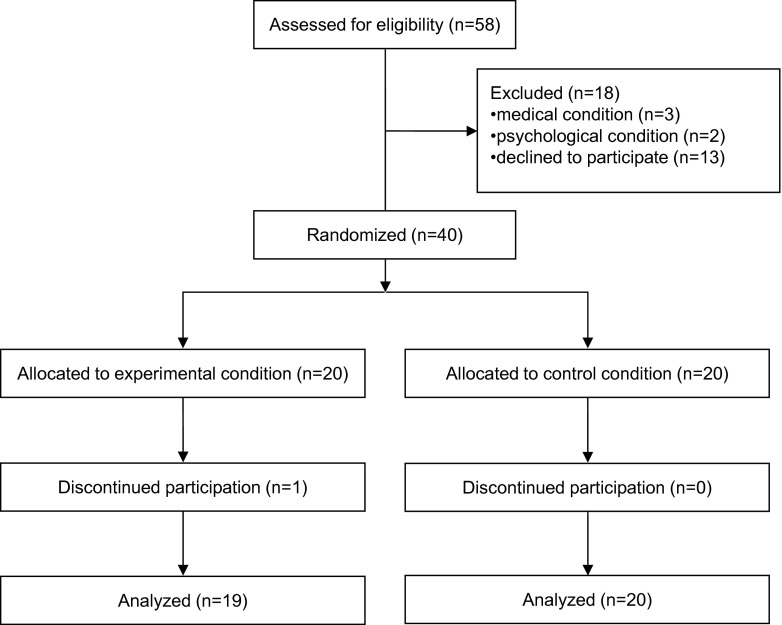
Flowchart

Forty participants were recruited and randomized equally to the two groups. In the experimental group, one participant discontinued drug intake due to side effects and was therefore excluded from study participation. Nineteen subjects in the amitriptyline group and 20 subjects in the placebo control group were thus included in our analyses (see Fig. 2). There were no significant differences in age, sex, or weight between participants in the two groups at baseline (see Table 1).

**Table 1:** Sample characteristics

Characteristics	Amitriptyline (*n* = 19)	Placebo (*n* = 20)	Group differences
Age in years, *M* (SD)	24.4 (3.5)	23.6 (3.7)	*t* (37) = −0.71, *p* = .481
Number females, *n* (%)	11 (57.9)	11 (55.0)	*χ* ^2^ (1) = 0.03, *p* = .556
Weight in kg, *M* (SD)	67.5 (11.3)	63.9 (9.0)	*t* (36^a^) = −1.09, *p* = .285

^a^One participant in the placebo group did not answer this question

### Primary outcome—antidepressant-specific side effects

#### GASE-AD (antidepressant-specific side effects)

**Fig. 3:**
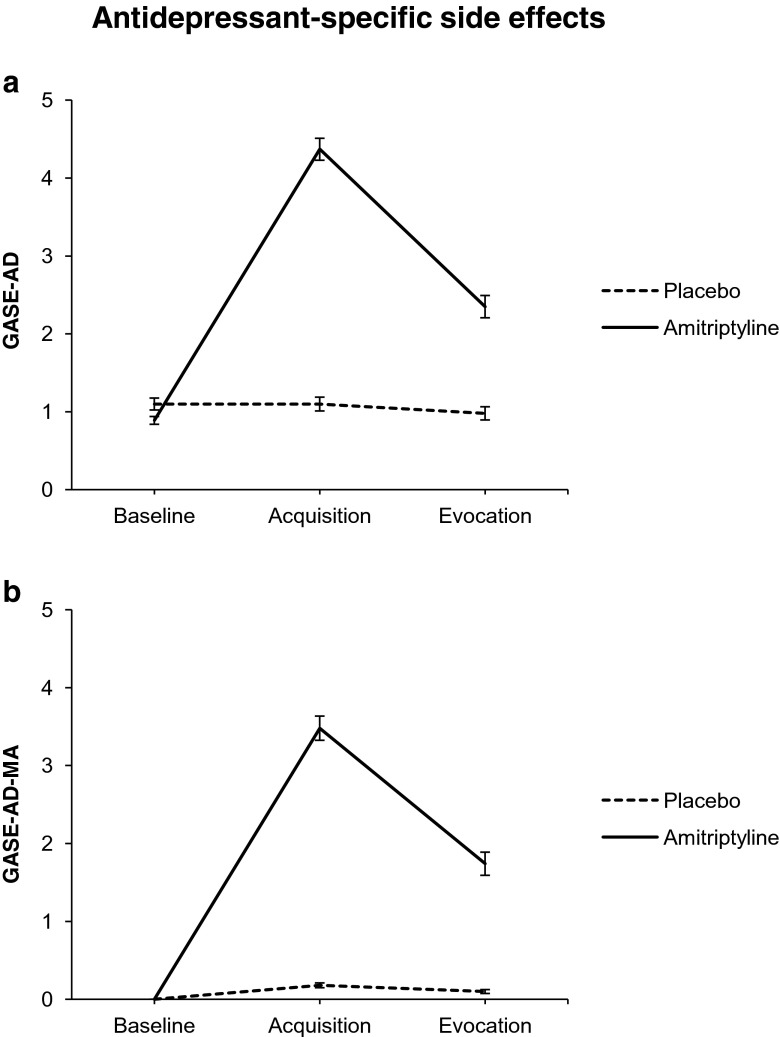
Antidepressant-specific side effects for both groups and all time points. *GASE-AD* Antidepressant Composite Score of the Generic Assessment of Side Effects Scale, *GASE-AD-MA* medication-attributed symptoms of the Antidepressant Composite Score of the Generic Assessment of Side Effects Scale

Overall multivariate analyses offered the basis for subsequent pairwise comparisons of single conditions (group effect *F* (2, 36) = 13.26, *p* ≤ .001; time effect *F* (4, 34) = 10.33, *p* ≤ .001; group × time interaction effect *F* (4, 34) = 8.17, *p* ≤ .001; univariate analyses: group effect *F* (1, 37) = 11.27, *p* = .002; time effect *F* (1, 37) = 14.57, *p* ≤ .001; group × time interaction effect *F* (1, 37) = 14.37, *p* ≤ .001). We observed that the two groups differed significantly in reported antidepressant-specific side effects after the acquisition phase (*p* ≤ .001; effect size Hedge’s *g* = 1.56; 95 % confidence interval (CI) 0.84–2.28) and after the evocation night (*p* = .045; *g* = 0.66; CI 0.01–1.30); the amitriptyline group reported significantly more side effects (see Table 2 and Fig. 3a). Furthermore, the amitriptyline group displayed significant differences between baseline and acquisition (*p* ≤ .001), between baseline and evocation (*p* = .007), and between acquisition and evocation (*p* ≤ .001). After the acquisition phase, subjects in the experimental group reported significantly more side effects compared with baseline and evocation. After the evocation night, participants also reported significantly more side effects compared with baseline.

**Table 2:** Means, standard deviations, and *F*-statistics for the univariate analyses for the different side effect scores

	Amitriptyline *M* (SD)	Placebo *M* (SD)	Time effect	Group effect	Interaction
Primary outcome					
GASE-AD			*F* (1, 37) = 14.57**	*F* (1, 37) = 11.27*	*F* (1, 37) = 14.37**
Baseline	0.89 (0.88)	1.10 (1.37)			
Acquisition	4.37 (2.45)	1.10 (1.59)			
Evocation	2.35 (2.49)	0.98 (1.51)			
GASE-AD-MA			*F* (1, 37) = 17.31**	*F* (1, 37) = 27.21**	*F* (1, 37) = 14.12**
Baseline	0 (0)	0 (0)			
Acquisition	3.48 (2.59)	0.18 (0.51)			
Evocation	1.74 (2.58)	0.10 (0.45)			
Further analyses					
GASE-generic			*F* (1, 37) = 0.84	*F* (1, 37) = 0.23	*F* (1, 37) = 2.05
Baseline	4.32 (3.68)	3.75 (3.68)			
Acquisition	5.63 (4.78)	3.85 (3.69)			
Evocation	3.68 (3.43)	4.42 (5.49)			
GASE-generic-MA			*F* (0.60, 22.20)^a^ = 5.43*	*F* (0.60, 22.20)^a^ = 3.88	*F* (0.60, 22.20)^a^ = 2.87
Baseline	0 (0)	0 (0)			
Acquisition	1.87 (3.40)	0.37 (1.09)			
Evocation	0.48 (0.91)	0.21 (0.90)			
GASE-total			*F* (0.85, 31.48)^a^ = 5.61*	*F* (0.85, 31.48)^a^ = 2.13	*F* (0.85, 31.48)^a^ = 6.11*
Baseline	5.21 (3.55)	4.85 (4.80)			
Acquisition	10.00 (5.24)	4.95 (4.95)			
Evocation	6.03 (4.58)	5.40 (6.77)			
GASE-total-MA			*F* (0.76, 22.28)^a^ = 14.53**	*F* (0.76, 22.28)^a^ = 23.45**	*F* (0.76, 22.28)^a^ = 10.04*
Baseline	0 (0)	0 (0)			
Acquisition	5.36 (4.69)	0.55 (1.57)			
Evocation	2.22 (3.17)	0.31 (1.34)			
GASE-AMI			*F* (1, 37) = 14.52**	*F* (1, 37) = 9.13*	*F* (1, 37) = 15.08**
Baseline	1.05 (1.13)	1.40 (1.89)			
Acquisition	5.33 (2.91)	1.35 (2.11)			
Evocation	2.87 (2.84)	1.28 (2.41)			
GASE-AMI-MA			*F* (1, 37) = 17.01**	*F* (1, 37) = 31.54**	*F* (1, 37) = 14.98**
Baseline	0 (0)	0 (0)			
Acquisition	4.06 (2.86)	0.18 (0.51)			
Evocation	1.89 (2.88)	0.10 (0.45)			

*GASE-AD* Antidepressant Composite Score of the Generic Assessment of Side Effects Scale, *GASE-AD-MA* medication-attributed symptoms of the Antidepressant Composite Score of the Generic Assessment of Side Effects Scale, *GASE-generic* generic symptoms on the Generic Assessment of Side Effects Scale, *GASE-generic-MA* medication-attributed generic symptoms on the Generic Assessment of Side Effects Scale, *GASE-total* all reported side effects as assessed with the Generic Assessment of Side Effects Scale, *GASE-total-MA* all medication-attributed side effects as assessed with the Generic Assessment of Side Effects Scale, *GASE-AMI* score of all common side effects of amitriptyline, *GASE-AMI-MA* score of all medication-attributed common side effects of amitriptyline

*p ≤ .05

**p ≤ .001

^a^Degrees of freedom have been corrected according to Greenhous-Geisser

#### GASE-AD-MA (medication-attributed antidepressant-specific side effects)

We found that the amitriptyline group’s medication-attributed, antidepressant-specific side effect score was significantly higher after the acquisition phase (*p* ≤ .001; *g* = 1.75; CI 1.02–2.49) and after the evocation night (*p* = .008; *g* = 0.88; CI 0.22–1.54) than the placebo group’s (see Table 2 and Fig. 3b). We also noted significant within-group differences between both baseline and acquisition (*p* ≤ .001) and between baseline and evocation (*p* = .001) on the GASE-AD-MA in the amitriptyline group. Thus, more antidepressant-specific side effects were attributed to the medication after the acquisition phase (i.e., intake of amitriptyline for four nights) than at baseline. More importantly, however, more antidepressant-specific side effects were also reported as medication-attributed after evocation night (i.e., after placebo intake) than at baseline. Furthermore, the amitriptyline group also reported significantly more medication-attributed symptoms after the acquisition phase than after the evocation night (*p* = .006; univariate analyses: group effect *F* (1, 37) = 27.21, *p* ≤ .001; time effect *F* (1, 37) = 17.31, *p* ≤ .001; group × time interaction effect *F* (1, 37) = 14.12, *p* ≤ .001).

### Further analyses

#### GASE-generic (generic side effects)

Our results reveal that only in the amitriptyline group, there was a significant difference between baseline and acquisition phase in medication-attributed generic side effects (GASE-generic-MA; *p* = .007). Participants attributed more generic symptoms to the medication after the acquisition phase than at baseline. We observed no differences between the groups in either the GASE-generic or GASE-generic-MA (multivariate analyses: group effect *F* (2, 36) = 1.89, *p* = .166; time effect *F* (4, 34) = 2.83, *p* = .040; group × time interaction effect *F* (4, 34) = 2.19, *p* = .091; univariate analyses regarding GASE-generic-MA: time effect *F* (0.60, 22.20) = 5.43, *p* = .027).

#### GASE-total (all side effects)

The experimental group reported significantly more total side effects (GASE-total) at acquisition than the control group (*p* = .003), and it reported significantly more medication-attributed total side effects (GASE-total-MA) at acquisition (*p* ≤ .001) and at evocation (*p* = .018). Only for the experimental group, significant within-group differences between the assessment points could be observed for the GASE-total score between baseline and acquisition (*p* ≤ .001) and between acquisition and evocation (*p* = .008). For the GASE-total-MA, significant differences between all assessment points could be observed in the experimental group (multivariate analyses: group effect *F* (2, 36) = 11.46, *p* ≤ .001; time effect *F* (4, 34) = 8.80, *p* ≤ .001; group × time interaction effect *F* (4, 34) = 6.75, *p* ≤ .001; for detailed results of the univariate analyses, see Table 2).

#### GASE-AMI (common side effects of amitriptyline)

Pairwise comparisons showed that there were significant group differences at acquisition in the GASE-AMI score (*p* ≤ .001) with the experimental group reporting more side effects. In the medication-attributed score for common side effects of amitriptyline (GASE-AMI-MA), groups differed significantly at acquisition (*p* ≤ .001) and at evocation (*p* = .009) in the direction that the experimental group reported more side effects. For the experimental group, within-group differences were significant for comparisons between all time points on the GASE-AMI and on the GASE-AMI-MA (multivariate analyses: group effect *F* (2, 36) = 15.35, *p* ≤ .001; time effect *F* (4, 34) = 11.61, *p* ≤ .001; group × time interaction effect *F* (4, 34) = 9.89, *p* ≤ .001; for detailed results of the univariate analyses, see Table 2).

#### Perceived group allocation

Group allocation as rated subjectively by the participants after the acquisition phase (perceived group allocation) correlated significantly with actual group allocation (*φ* = .641), meaning that 82 % of participants guessed their group allocation correctly, indicating the participants’ at least partial unblinding to group allocation.

#### Nocebo response correlates

We detected no significant correlations among either the SCL-90-R subscales or BDI and the GASE-AD and GASE-AD-MA at any time point (baseline, acquisition, or evocation) in the amitriptyline group, indicating the nocebo response’s independence of these clinical features.

## Discussion

The aim of our study was to investigate whether antidepressant-specific side effects are not only caused by the drug’s pharmacological actions but also learned through classical conditioning. We found that antidepressant-specific side effects can be evoked by an identical-looking placebo pill in participants who had previously taken an antidepressant that was accompanied by the same stimulus (novel-tasting drink) as the intake of placebo. In addition, participants who had previously been taking the antidepressant rated more of the side effects after taking the placebo pill as being medication-induced than participants who had been taking the placebo all the time. In contrast to the antidepressants’ specific side effects, the generic side effect score did not change significantly between the assessment points, although participants taking amitriptyline attributed more of these generic side effects to medication intake after acquisition.

These findings suggest that learning plays a role in the experiencing and reporting of side effects from antidepressants. This result is highly relevant, since patients suffering from depression often experience several depressive episodes in their lives and usually undergo repeated pharmacological treatment (Solomon and Keller 2000). There is evidence that patients who have been prescribed antidepressant medication once are more likely to be prescribed antidepressants again (Sirey and Meyers 2014). Our results suggest that if a participant has had negative experiences with a certain drug before, learning processes may contribute to the re-occurrence of these side effects. This in turn may lead to non-adherence or drug discontinuation (e.g., Serna et al. 2010; Hung et al. 2011; Murata and Kanbayashi 2012; De las Cuevas et al. 2014) and hence to a worse outcome or higher risk of relapses (Åkerblad et al. 2006). Given that side effects seem to depend on prior experience, it would seem advisable to systematically assess a patient’s prior experience with specific drugs before issuing a prescription and in case of negative experiences to try another drug (Doering and Rief 2013).

Our finding that certain side effects can be learned is in line with research showing that learning plays an important role in nocebo effects (e.g., Colloca et al. 2008; Klosterhalfen et al. 2009). Furthermore, it also falls in line with studies demonstrating that pharmacological responses can be conditioned (Goebel et al. 2008; Attwood et al. 2010; Albring et al. 2012). There have been proposals and even tests involving conditioning procedures to reduce drug doses in pharmacotherapy, a mechanism called placebo-controlled dose reduction (Ader et al. 2010; for a review, see Doering and Rief 2012). The maintenance of a drug’s therapeutic effect while possibly reducing side effects and hence enhancing compliance has been postulated as one advantage of placebo-controlled dose reduction (Doering and Rief 2012). However, both the placebo effect of drug intake can obviously be learned, as can the nocebo effect, a factor that should be considered when planning placebo-controlled dose reduction.

Participants taking amitriptyline attributed more generic symptoms to medication intake, although their generic-symptom score after acquisition was not significantly higher than their own baseline and the placebo group’s scores, revealing that part of the side effects patients report may be due to the misattribution of pre-existing symptoms. By thoroughly assessing symptoms and side effects including baseline evaluations (Rief et al. 2006), we have succeeded in demonstrating this “misassignment” of symptom attribution. This is an already-described phenomenon (Barsky et al. 2002). The effect in our study was admittedly rather small and needs to be replicated. However, it is an extremely relevant phenomenon in pharmacotherapy since the attribution of side effects to the medication is an important factor behind the discontinuation of medication intake.

Some shortcomings of the present study should be mentioned. First, we only assessed subjective data as outcome measures. Participants may have reported more symptoms in general because they were taking a drug, even though they did not attribute them to that drug. To account for this bias, we differentiated between reported symptoms in general and medication-attributed symptoms. In addition, the structured assessment of side effects (rather than an unstructured evaluation) may trigger more reported side effects (Rief et al. 2006). Secondly, the correlation between perceived group allocation and actual group allocation indicates that at least some participants were unblinded to group allocation, a problem reported and discussed in previous antidepressant trials in general (Jeffrey et al. 1986; Margraf et al. 1991). In terms of our study, this might imply that the unblinding shaped the participants’ expectations regarding the pill in the evocation night, with the amitriptyline group expecting more side effects. Thirdly, the generalizability of our results to a clinical setting is limited since we only examined healthy young individuals and always paired the drug intake with a salient new stimulus. Hence, we cannot conclude whether a paradigm in which only the pill’s appearance (without a salient new stimulus) in the typical treatment context serves as the conditioned stimulus (the case in natural clinical settings) would evoke the same amount of side effects. A fourth limitation is that since this study was just a pilot trial, we did not incorporate an untreated group in the study design, something other researchers suggest (Colloca and Miller 2011). Finally, it is a shortcoming that it would have been advantageous to include an assessment of side effects after the washout phase in order to control for any residual symptoms from the acquisition phase. One could argue that the side effects occurring in the evocation night were only due to a residual concentration of amitriptyline in the blood. However, there is solid evidence that the plasma half-life of tricylic antidepressants ranges from 10 to 28 h (Rudorfer and Potter 1999). Our washout phase entailed three nights without medication intake, which is 86 h between the last intake of amitriptyline and intake of placebo and should rule out the argument that a residual concentration of amitriptyline might have accounted for the difference in the evocation night. In addition, our participants received only low doses of amitriptyline (50 mg). Nevertheless, one could also argue that the antidepressant-specific side effects reported in the evocation night were due to discontinuation effects of amitriptyline, which might include gastrointestinal symptoms, affective symptoms, general somatic symptoms, and sleep disturbance (Haddad and Anderson 2007). However, the longer the treatment lasts and the higher the dosage of antidepressant, the more likely discontinuation symptoms occur (Kramer et al. 1961; Perahia et al. 2005). Both of these circumstances do not apply to our study, meaning that the explanation that differences between the amitriptyline and placebo group at the evocation night were only due to discontinuation effects is not likely.

Despite these limitations, our study encourages future research to examine Pavlovian conditioning in conjunction with side effects. To learn more about these mechanisms, future studies should vary the number of learning trials and of the intervals between acquisition and evocation. It would be critical to determine if and when conditioned effects extinguish when the interval between acquisition and evocation is long enough. In addition, we only examined the effect associated with one drug; thus, the learning of side effects should also be addressed in conjunction with other drugs. To draw conclusions for pharmacotherapy in clinical settings, patients rather than healthy individuals need to be examined. Understanding the mechanisms that lead to the learning of side effects may help to prevent the side effects triggered by prior experience. Several proposals about how to reduce side effects have been made (Colloca and Miller 2011; Bingel 2014). Such interventions focus mainly on the expectations of side effects induced by verbal suggestion or other information and how to modify them to minimize side effects (von Blanckenburg et al. 2013). As our findings suggest that prior negative experience with a drug can also lead to side effects and hence non-adherence, it is important to develop additional strategies to prevent these learned side effects.

## References

[CR1] Abbing-Karahagopian V, Huerta C, Souverein PC (2014). Antidepressant prescribing in five European countries: application of common definitions to assess the prevalence, clinical observations, and methodological implications. Eur J Clin Pharmacol.

[CR2] Ader R, Mercurio MG, Walton J (2010). Conditioned pharmacotherapeutic effects: a preliminary study. Psychosom Med.

[CR3] Åkerblad A-C, Bengtsson F, von Knorring L, Ekselius L (2006). Response, remission and relapse in relation to adherence in primary care treatment of depression: a 2-year outcome study. Int Clin Psychopharmacol.

[CR4] Albring A, Wendt L, Benson S (2012). Placebo effects on the immune response in humans: the role of learning and expectation. PLoS One.

[CR5] Amanzio M (2015). Nocebo effects and psychotropic drug action. Expert Rev Clin Pharmacol.

[CR6] Amanzio M, Corazzini LL, Vase L, Benedetti F (2009). A systematic review of adverse events in placebo groups of anti-migraine clinical trials. Pain.

[CR7] Ashton AK, Jamerson BD, Weinstein WL, Wagoner C (2005). Antidepressant-related adverse effects impacting treatment compliance: results of a patient survey. Curr Ther Res Clin Exp.

[CR8] Attwood A, Terry P, Higgs S (2010). Conditioned effects of caffeine on performance in humans. Physiol Behav.

[CR9] Barnhart ER (1988). Physician’s desk reference, 42nd edn.

[CR10] Barsky A, Saintfort R, Rogers M, Borus J (2002). Nonspecific medication side effects and the nocebo phenomenon. JAMA.

[CR11] Beck A, Ward C, Medelson M (1961). An inventory for measuring depression. Arch Gen Psychiatry.

[CR12] Benkert O, Hippius H (2014). Kompendium der psychiatrischen Pharmakotherapie.

[CR13] Bingel U (2014). Avoiding nocebo effects to optimize treatment outcome. JAMA.

[CR14] Bocquier A, Cortaredona S, Verdoux H (2014). Social inequalities in early antidepressant discontinuation. Psychiatr Serv.

[CR15] Cleare A, Pariante CM, Young AH (2015). Evidence-based guidelines for treating depressive disorders with antidepressants: a revision of the 2008 British Association for Psychopharmacology guidelines. J Psychopharmacol.

[CR16] Cohen S (2014). The nocebo effect of informed consent. Bioethics.

[CR17] Colloca L, Miller FG (2011). The nocebo effect and its relevance for clinical practice. Psychosom Med.

[CR18] Colloca L, Sigaudo M, Benedetti F (2008). The role of learning in nocebo and placebo effects. Pain.

[CR19] Colloca L, Petrovic P, Wager T (2010). How the number of learning trials affects placebo and nocebo responses. Pain.

[CR20] De las Cuevas C, Peñate W, Sanz EJ (2014). Risk factors for non-adherence to antidepressant treatment in patients with mood disorders. Eur J Clin Pharmacol.

[CR21] Derogatis LR (1994). Symptom checklist-90-revised: administration, scoring and procedures manual.

[CR22] Doering BK, Rief W (2012). Utilizing placebo mechanisms for dose reduction in pharmacotherapy. Trends Pharmacol Sci.

[CR23] Doering BK, Rief W (2013) Nocebos in daily clinical practice. In: Colloca L, Flaten MA, Meissner K (eds) Placebo and pain: from bench to bedside. Academic Press, pp 257–266

[CR24] Enck P, Bingel U, Schedlowski M, Rief W (2013). The placebo response in medicine: minimize, maximize or personalize?. Nat Rev Drug Discov.

[CR25] Faul F, Erdfelder E, Lang A-G, Buchner A (2007). G*power 3: a flexible statistical power analysis program for the social, behavioral, and biomedical sciences. Behav Res Methods.

[CR26] Goebel MU, Meykadeh N, Kou W (2008). Behavioral conditioning of antihistamine effects in patients with allergic rhinitis. Psychother Psychosom.

[CR27] Haddad PM, Anderson IM (2007). Recognising and managing antidepressant discontinuation symptoms. Adv Psychiatr Treat.

[CR28] Hiller W, Zaudig M, Mombour W (2004). Internationale Diagnose Checklisten für ICD-10 und DSM-IV.

[CR29] Hung C-I, Wang S-J, Liu C-Y (2011). Comorbidities and factors related to discontinuation of pharmacotherapy among outpatients with major depressive disorder. Compr Psychiatry.

[CR30] Jeffrey GR, Harrison W, Quitkin FM, Klein DF (1986). How blind is blind? Assessment of patient and doctor medication guesses in a placebo-controlled trial of imipramine and phenelzine. Psychiatry Res.

[CR31] Jensen KB, Kaptchuk TJ, Kirsch I (2012). Nonconscious activation of placebo and nocebo pain responses. Proc Natl Acad Sci U S A.

[CR32] Klosterhalfen S, Kellermann S, Braun S (2009). Gender and the nocebo response following conditioning and expectancy. J Psychosom Res.

[CR33] Kramer JC, Klein DF, Fink M (1961). Withdrawal symptoms following discontinuation of imipramine therapy. Am J Psychiatry.

[CR34] Lockhart P, Guthrie B (2011) Trends in primary care antidepressant prescribing 1995–2007. Br J Gen Pract:565–572. doi:10.3399/bjgp11X593848.e565 10.3399/bjgp11X593848 PMC3162179 22152736

[CR35] Margraf J, Ehlers A, Roth WT (1991). How “blind” are double-blind studies?. J Consult Clin Psychol.

[CR36] Matteson S, Roscoe J, Hickok J, Morrow GR (2002). The role of behavioral conditioning in the development of nausea. Am J Obstet Gynecol.

[CR37] Mitsikostas DD (2012). Nocebo in headaches: implications for clinical practice and trial design. Curr Neurol Neurosci Rep.

[CR38] Mojtabai R, Olfson M (2014). National trends in long-term use of antidepressant medications. J Clin Psychiatry.

[CR39] Mondaini N, Gontero P, Giubilei G (2007). Finasteride 5 mg and sexual side effects: how many of these are related to a nocebo phenomenon?. J Sex Med.

[CR40] Murata A, Kanbayashi T (2012) Risk factors for drug nonadherence in antidepressant-treated patients and implications of pharmacist adherence instructions for adherence improvement. Patient Prefer Adherence 863–869 10.2147/PPA.S36295 PMC3526883 23271895

[CR41] Nestoriuc Y, Orav EJ, Liang MH (2010). Prediction of nonspecific side effects in rheumatoid arthritis patients by beliefs about medicines. Arthritis Care Res (Hoboken).

[CR42] Pavlov IP (2010). Conditioned reflexes: an investigation of the physiological activity of the cerebral cortex. Ann Neurosci.

[CR43] Perahia DG, Kajdasz DK, Desaiah D, Haddad PM (2005). Symptoms following abrupt discontinuation of duloxetine treatment in patients with major depressive disorder. J Affect Disord.

[CR44] Rief W, Avorn J, Barsky AJ (2006). Medication-attributed adverse effects in placebo groups—implications for assessment of adverse effects. Arch Intern Med.

[CR45] Rief W, Nestoriuc Y, von Lilienfeld-Toal A (2009). Differences in adverse effect reporting in placebo groups in SSRI and tricyclic antidepressant trials. Drug Saf.

[CR46] Rief W, Barsky AJ, Glombiewski JA (2010). Assessing general side effects in clinical trials: reference data from the general population. Pharmacoepidemiol Drug Saf.

[CR47] Rudorfer M, Potter W (1999). Metabolism of tricyclic antidepressants. Cell Mol Neurobiol.

[CR48] Sawada N, Uchida H, Suzuki T (2009). Persistence and compliance to antidepressant treatment in patients with depression: a chart review. BMC Psychiatry.

[CR49] Schedlowski M, Enck P, Rief W, Bingel U (2015). Neuro-bio-behavioral mechanisms of placebo and nocebo responses: implications for clinical trials and clinical practice. Pharmacol Rev.

[CR50] Serna MC, Cruz I, Real J (2010). Duration and adherence of antidepressant treatment (2003 to 2007) based on prescription database. Eur Psychiatry.

[CR51] Shedden Mora M, Nestoriuc Y, Rief W (2011). Lessons learned from placebo groups in antidepressant trials. Philos Trans R Soc Lond Ser B Biol Sci.

[CR52] Sihvo S, Wahlbeck K, Mccallum A (2010). Increase in the duration of antidepressant treatment from 1994 to 2003: a nationwide population-based study from Finland. Pharmacoepidemiol Drug Saf.

[CR53] Sirey J, Meyers B (2014). Predictors of antidepressant prescription and early use among depressed outpatients. Am J Psychiatry.

[CR54] Solomon D, Keller M (2000). Multiple recurrences of major depressive disorder. Am J Psychiatry.

[CR55] Stewart-Williams S, Podd J (2004). The placebo effect: dissolving the expectancy versus conditioning debate. Psychol Bull.

[CR56] von Blanckenburg P, Schuricht F, Albert U-S (2013). Optimizing expectations to prevent side effects and enhance quality of life in breast cancer patients undergoing endocrine therapy: study protocol of a randomized controlled trial. BMC Cancer.

[CR57] Winkler A, Rheker J, Doering BK, Rief W (2016). Conditioning of amitriptyline-induced REM sleep suppression in healthy participants: a randomized controlled trial. Psychophysiology.

[CR58] (2015) http://www.pharmawiki.ch. http://www.pharmawiki.ch.

